# Ultra percutaneous dilation tracheotomy vs mini open tracheotomy. A comparison of tracheal damage in fresh cadaver specimens

**DOI:** 10.1186/s13104-015-1199-4

**Published:** 2015-06-10

**Authors:** Khalid AL-Qahtani, Jon Adamis, Jennifer Tse, Jeffery Harris, Tahera Islam, Hadi Seikaly

**Affiliations:** Division of Otolaryngology-Head and Neck Surgery, University of Alberta, Edmonton, Canada; Department of Otolaryngology-Head and Neck Surgery, College of Medicine, King Abdul Aziz University Hospital, King Saud University, PO Box no-245, Riyadh, 11411 Kingdom Saudi Arabia; College of Medicine and Research Center, King Saud University, Riyadh, Kingdom Saudi Arabia

**Keywords:** Ultra percutaneous dilation tracheostomy (UPDT), Mini open tracheostomy (MOT), Airway management, Tracheal stenosis

## Abstract

**Background:**

To compare the ultra percutaneous dilation tracheostomy (PDT) and mini open techniques (MOT) in randomized fixed and fresh cadavers. Assess degrees of damage to tracheal cartilage and mucosa via tracheal lumen and external dissection.

**Method:**

Comparative cadaver study was performed, tracheostomy was placed in 36 cadavers (16 fixed, 20 fresh) from July 2004 to December 2004, in University of Alberta, Canada. PDT (*size 7*) were placed by intensivist and MOT (size 7) otolaryngologist. Both fixed and fresh cadavers were randomized. Evaluation was done according to gender, ease of landmark, mucosal and cartilage injuries.

**Results:**

Significant differences in mucosal injury (7 of 9 in UPDT VS 0 of 7 in MOT, p value 0.008), and cartilage injury (8 of 9 in UPDT VS 1 of 7 in MOT p value 0.012) were seen in fixed cadavers; and in fresh cadavers, mucosal injury (5 of 10 in UPDT VS 0 of 10 in MOT, p value 0.043), and cartilage injury (5 of 10 in UPDT VS 0 of 10 in MOT, p value 0.043).

**Conclusions:**

PDT resulted in severe damage to mucosa and cartilage, that might contribute to subglottic stenosis preventing decannulation. Considering the injury, MOT has better outcome than UPDT.

## Background

Tracheostomy is a common surgical procedure [1], first defined in modern medicine by Jackson [2] in 1909 and was performed primarily for airway stenosis. As modern intensive care developed, the functionality of tracheostomy shifted to long-term ventilation and management of pulmonary secretions [1]. The indications for a tracheostomy include anticipated prolonged respiratory assistance, access to lower airway secretions and the prevention of injury to larynx [3].

Within the last two decades, open surgical tracheostomy has been increasingly replaced by percutaneous dilational tracheostomy [4]. Sheldon et al. [5] performed the first trials of bedside percutaneous tracheostomy in 1955, but it was not widely used until Ciaglia et al. [6] described the first percutaneous dilational tracheostomy (PDT) in 1985. Minimal complication rates, efficiency, cost-effectiveness [5, 7, 8] and the convenience of the bedside procedure [6, 9, 10] have resulted in widespread use of this technique.

The introduction of flexible bronchoscopy has made tracheostomy easy to teach with minor intraoperative complications in the hands of an experienced team [11, 12].

Much less is reported about the potential late complications of dilational tracheostomy compared with the surgical technique [8, 13]. Several studies have compared the short- and long-term outcomes of open tracheostomy with PDT [14–16]. Short-term benefits of PDT over open tracheostomy in the uncomplicated patient are decreased hemorrhage at the wound site, less infection, decreased tube displacement, and lower costs [17, 18]. Long-term complications suggested a decrease in tracheal stenosis [19].

The commonest arguments in favour of dilational tracheostomy are reduced operative time, ease of performance, ability to be performed at the bedside and lower cost [20, 21]. However, if flexible bronchoscopy is performed, both procedures require sedation, special equipment and assistance, bringing into question some of the advantages proposed for dilational tracheostomy [22]. In fact, all tracheostomy techniques can be performed at the bedside with relative ease [18]. Nevertheless, the perceived advantages of dilational techniques make this procedure the first choice [23] for the critically ill patient needing long-term airway control [24].

Increasing number of patients with a tracheostoma are discharged from the intensive care unit prior to decannulation. Few intensive care services carry out long-term follow-up of these patients [23]. Recent case reports, suggest that there may be a high incidence of suprastomal and subglottic tracheal stenosis as a late complication after dilational tracheostomy [25, 26].

Several theories relating to causes of tracheal stenosis after PDT have been proposed in the literature, including aberrant placement of the tracheostomy and increased insertional pressure on the cartilage [1]. In a series by Norwood et al. [13], 11 (31%) of 422 long-term PDT patients had more than 10% stenosis documented by tracheal computed tomography (CT), and 2% had severe stenosis reducing the tracheal diameter by >50%. All areas of stenosis in the study of Norwood et al. [13] occurred at the stoma site. These studies prompted a preliminary cadaveric study to evaluate the anatomical effects of PDT at the stoma and surrounding insertion site for a common injury pattern that may contribute to tracheal stenosis.

Method of tracheotomy has been the subject of much research and debate. Endoscopically aided placement has improved accuracy. Newer developments in PDT kits promise to minimize complications.

This study was designed to compare the ultra PDT and mini open techniques in randomized fixed and fresh cadavers, also to assess degrees of damage to tracheal cartilage and mucosa via tracheal lumen and external dissection.

## Methods

A comparative cadaver study was performed in which tracheostomy was placed in thirty six cadavers from July 2004 to December 2004, in University of Alberta, Canada. The study was approved by the human research ethics board at the University of Alberta, Canada. Sixteen cadavers were preserved according to formalin embalming method and 20 were fresh. The fresh cadavers were from persons who had died <48 h previously before the study. Ultra percutaneous dilation tracheostomy (Portex Blue Line *Ultra*, *size 7*) were done by an experienced intensivist. Mini open tracheostomy (size 7) were done by an experienced otolaryngologist. The 16 fixed cadavers were randomized to be done either by the UPDT or MOT, and the same done for the fresh cadavers. Cadaver features evaluated included gender, and ease of landmark identification. Mucosal and cartilage injuries were evaluated on of Assigned an Injury Score (0 or 1). Then the data were analyzed by blinded examiner.

Once fixed, there was very little change. Also, mucosa and cartilage became very brittle and no bleeding occurred. The fresh cadavers (n = 20) were preserved <48 h. Mucosa and cartilage were unchanged (more resilient). They have vascular backflow. Also, they have similar properties of skin, fascia and thyroid. Characteristics of each cadaver were analyzed and recorded at the time of tracheostomy placement, including gender, adequacy of anatomical landmarks (cricoid cartilage, thyroid notch, sternal notch), and ease of neck extension. Subjective assessment of stoma site placement, degree of surrounding tissue injury, and degree of cartilage injury were conducted.

**Figure 1:**
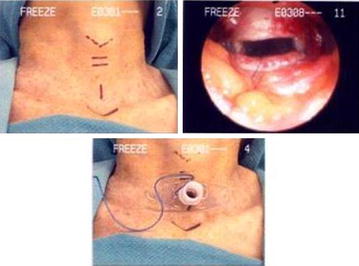
Mini open tracheostomy-preoperative surface mark of the incisions and operative appearance and final result tracheostomy in place.

An open mini tracheostomy was performed on seven fixed cadaver and ten fresh cadaver. A 1 cm vertical incision was used and positioned 1 cm below the cricoid cartilage (Figure 1). The strap muscles were separated, and the thyroid isthmus was divided. The trachea was entered via a horizontal incision above the second or third tracheal ring. An inferiorly based anterior tracheal wall flap was created by cutting the tracheal ring laterally, and a stay silk suture was placed through the tracheal wall flap. A seven trach tube was used.

**Figure 2:**
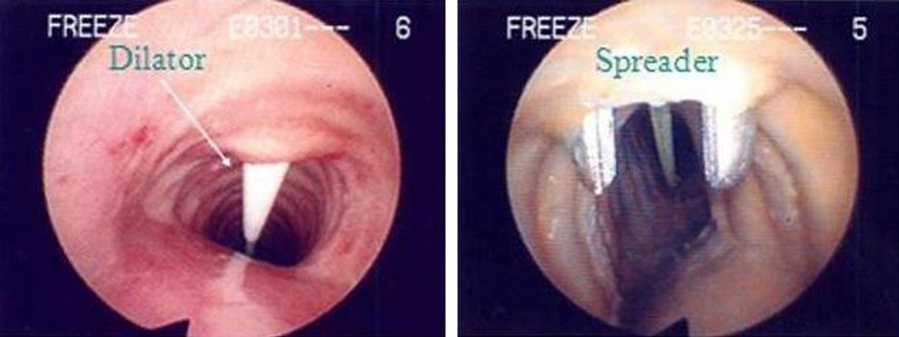
Percutaneous dilatational tracheostomy technique.

Percutaneous dilational tracheostomy was performed by an experienced intensivist on nine fixed cadaver and ten fresh cadaver using a commercially available kit (Portex Blue Line *Ultra*, *size 7*), following the technique described by Ciaglia [6] (Figure 2). After maximal extension of the neck, appropriate anatomical landmarks were palpated and identified. A 1.5-cm vertical midline incision was made over the trachea. Tissue was carefully dissected to the level of the tracheal cartilage identifying a tracheal interspace below the cricoids cartilage. A cannulated needle on a saline-filled syringe was used to puncture the trachea, with aspiration confirming placement in the lumen. A guidewire was introduced, serial dilations were undertaken, and the tracheostomy tube was introduced (Figure 2). After insertion, laryngotracheal specimens were harvested en bloc and analyzed by two otolaryngologists for injury at the stoma and surrounding tissue site.

Assessment of tracheal mucosal injury was graded on a scale of 0–4 as follows: 0 represented no appreciable mucosal tear beyond the intended stoma, 1 represented a mucosal tear into but not beyond one tracheal ring from the stoma, 2-a mucosal tear beyond one tracheal ring from the stoma, 3-bidirectional mucosal tears but not beyond the rings flanking the stoma, and 4-bidirectional mucosal tears beyond one tracheal ring flanking the stoma.

**Figure 3:**
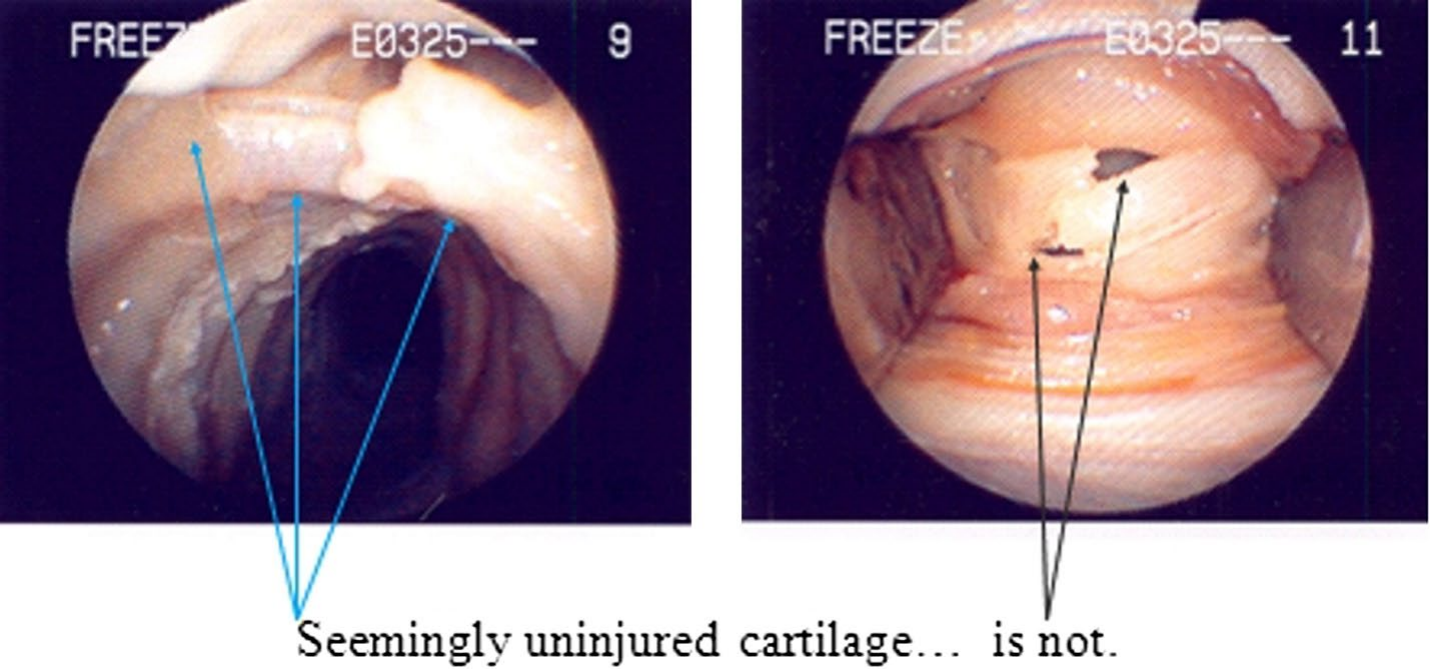
Different injuries to the tracheal rings.

**Figure 4:**
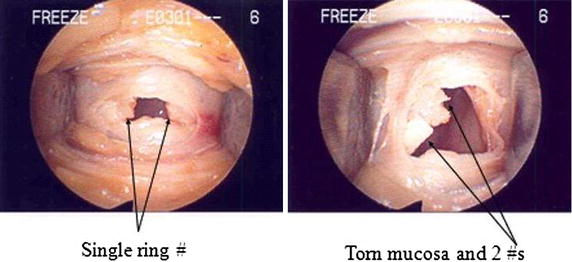
Different injuries to the tracheal rings.

Assessment of injury to the cartilaginous rings (Figures 3, 4) was evaluated on a scale of 0–4 as follows: 0 represented no appreciable cartilage fracture; 1-a cartilage fracture of one ring adjacent to the stoma, 2-a cartilage fracture of the superior and inferior error flanking the stoma, 3-a cartilage fracture into more that two cartilaginous rings, and 4-a fracture with multiple comminutions. A designation of “C” specified involvement of the cricoid cartilage.

The 16 fixed cadavers were randomized to be done either by the UPDT or MOT, and the same done for the fresh cadavers. Then the data were analyzed by blinded examiner, using olympus flexible laryngoscope and anatomical dissection. Statistical analysis was done using the modified grading system. In which, grade number corresponds to cartilage/mucosal ring damage. Expected injury if Grade <2, and Score of ‘0’. While beyond expected injury if Grade ≥2, and Score of ‘1’.

## Results

Thirty six cadavers, 16 fixed and 20 fresh were enrolled in the study. Table 1 shows the demographics of the cadavers.

**Table 1:** Confounders in fresh cadaver and fixed cadavers

Confounder	Fresh cadaver		Fixed cadavers	
	PDT (n = 10)	Open (n = 10)	PDT (n = 9)	Open (n = 7)
Age	Mean = 79	Mean = 74	Mean = 81.5	Mean = 75.8
	Range = 69–74	Range = 62–88	Range = 68–97	Range = 65–88
Landmarks	Easy = 6	Easy = 5	Easy = 5	Easy = 5
	Difficult = 4	Difficult = 5	Difficult = 4	Difficult = 2
Sex	M = 5	M = 3	M = 5	M = 3
	F = 5	F = 7	F = 4	F = 4

Among the fresh cadavers, ten were in the PDT arm and ten in the MOT arm. The fresh cadavers in the PDT arm, five were male and five were female. The mean age of the fresh cadavers in the PDT arm was 79 years, ranging between 69 and 94 years. The landmark was easily identified in six cadavers, while it was difficult in four cadavers. Among the fresh cadavers in the MOT arm, 3 were male and 7 female. The mean age of the fresh cadavers in the MOT arm was 74 years, ranging between 62 and 88 years. The landmark was easily identified in five cadavers, while it was difficult in five cadavers.

There were sixteen fixed cadaver, with nine fixed cadavers in the PDT arm and seven fixed cadavers in the MOT arm. The fixed cadavers in the PDT arm, five were male and four were female. The mean age of the fixed cadavers in the PDT arm was 81.5 years, ranging between 68 and 97 years. The landmark was easily identified in five cadavers, while it was difficult in four cadavers. The fixed cadavers in the MOT arm, three were male and four were female. The mean age of the fixed cadavers in the MOT arm was 75.8 years, ranging between 65 and 88 years. The landmark was easily identified in five cadavers, while it was difficult in two cadavers.

Sixteen fixed cadavers were included in the final analysis of the data, with nine fixed cadavers in the PDT arm and seven fixed cadavers in the MOT arm. The fixed cadavers demographics are compared in Table 2. Significant difference was seen in PDT arm and MOT arm groups in each of the demographic categories (Table 2). The mucosal injury was found in seven cadavers in PDT arm, while there was no mucosal injury in the MOT arm, which is statistically significant (p 0.008). Also, cartilage injury was found in eight cadavers in PDT arm, while there was one cartilage injury in the MOT arm, which is statistically significant (p 0.012).

**Table 2:** Fresh and fixed cadavers injury analysis

Injury	Fresh cadaver			Fixed cadavers		
	PDT (n = 10)	Open (n = 10)	p-value	PDT (n = 9)	Open (n = 7)	p-value
Mucosal injury	5	0	0.043	7	0	<0.008
Cartilage injury	5	0	0.043	8	1	>0.012

Twenty fresh cadavers were included in the final analysis of the data, with ten fresh cadavers in the PDT arm and ten fresh cadavers in the MOT arm. The fresh cadavers demographics are compared in Table 2. Significant difference was seen in PDT arm and MOT arm groups in each of the demographic categories (Table 2). Mucosal injury was found in five cadavers in PDT arm, while there was no mucosal injury in the MOT arm, which is statistically significant (p 0.043). Also, the cartilage injury was found in five cadavers in PDT arm, while there was no cartilage injury in the MOT arm, which is statistically significant (p 0.043).

## Discussion

Tracheotomy is a very common procedure at tertiary centers like the University of Alberta Hospital; about 120 cases were done per year by an otolaryngology team, while 250 cases were done per year in the ICU. Prolonged ventilation, pulmonary toilet and emergencies are common indications. Method of tracheotomy has been the subject of much research and debate. In recent years we have noticed a trend in the development of subglottic stenosis in percutaneously trached patients. Traditionally a surgical airway was chosen, but many studies advocate the safety and efficacy of the percutaneous dilation method. We hypothesized that mini open tracheotomy results in more predictable injury patterns to the stoma micro anatomical environment then endoscopically guided ultra PDT.

In 1985, Ciaglia re-introduced the technique of percutaneous dilational tracheostomy, which consists of the insertion of a guidewire through a puncture of the trachea, followed by several ‘dilators’ to create a sufficiently wide stoma [6]. Since then, the use of different variations of non-surgical dilational tracheostomies has dramatically increased [10, 17, 27]. At least four commercial sets for such ‘minimal invasive’ procedures are currently in use. In three of them, the cannula is inserted from outside. The other technique described by Fantoni and Ripamonti [9] in 1997 adopts a one-step procedure combining dilation and cannula insertion. The device is introduced through the larynx and has to be rotated in the trachea to reach the correct position.

Percutaneous dilational tracheostomy is a procedure widely accepted by intensive care physicians. Studies comparing the versatility of open surgical vs dilational tracheostomy techniques provide compelling evidence that the latter saves time and expense and is easy to perform at the bed side [28]. However, little is known about the potential long-term complications of percutaneous dilational tracheostomy, especially in patients with a persisting tracheostoma [26].

Tracheal stenosis after tracheostomy was observed cranial to the tracheostoma and consisted of scar and granulation tissue. We speculate that the curved shape of the dilators and the recommended downward orientation of the dilator tip in the trachea used to avoid injury to the posterior tracheal wall results in an inward tearing of tracheal cartilage and soft tissues above the dilation site and an outward tearing below the dilation site. The outward pressure on the caudal anterior wall and an inward pressure on the cranial anterior wall of the trachea persist by the tracheal cannula due to the angle and the caudal direction of the tracheostomy canal. Therefore the intrusion of the anterior wall might be fixed in this position by the pressure of the inserted cannula. This could explain the frequently observed suprastomal tracheal stenosis.

In some of the classical non-flap surgical techniques such as simple horizontal incision of the anterior tracheal wall, a similar inward tearing mechanism of the suprastomal anterior tracheal wall can be assumed. In fact, stenosis as a complication associated with tracheostomy was described at the end of the 19th and the beginning of the 20th century before the introduction of translaryngeal intubation [29–31]. In those cases the tracheostoma was usually created by a classical open tracheostomy technique without a flap or by the use of ‘tracheotom’ instruments which appear very similar to the dilators used in the percutaneous dilational technique today [26].

In contrast to dilational tracheostomy and non-flap surgical tracheostomy, a flap tracheostomy with an inverted U-flap of the anterior tracheal wall seems to minimise the risk for the development of a severe suprastomal tracheal stenosis. The creation of a stable and epithelialised tracheostoma may help to avoid inward tearing of the suprastomal anterior tracheal wall, thereby decreasing the risk for the development of a suprastomal tracheal stenosis as a long-term complication [26].

In assessing anticipated ease of tracheostomy placement with tracheostomy, damage did not always correlate with the initial surgical impression. In several instances, mucosal and cartilaginous involvement was opposite of suspicion [1]. In our study, the difficulty of identifying the surgical landmark was not correlated with the mucosal or cartilage injury. Also, the age and the sex did not appear to correlate with injury to the tracheal wall.

Dexter [32] performed a cadaver study of 20 specimens in which PDT was placed and macroscopic analysis of the trachea was performed. He found that only 9 of 20 catheters (45%) entered at the intended position, with 7 higher and 4 lower than intended. Hotchkiss [1] compared their data of perceived tracheostomy placement versus actual placement, they found that three of six catheters entered at the intended position, with one higher and two lower. This supports Dexter’s study demonstrating that approximately 50% of PDT catheters are actually placed where intended. Risk to injury of the cricoid cartilage is increased during high placement of PDT. Also, in Hotchkiss [1], the stoma site being placed higher than intended resulted in multiple comminuted fractures involving the cricoid cartilage. As suggested by Jackson [2] cricoid comminutions during PDT may contribute to postoperative subglottic and tracheal stenosis in vivo.

The theory of PDT placement proposed by Hazard et al. [33] suggests that PDT may cause less risk for tracheal stenosis after decannulation because the cartilaginous rings remain intact. Rather than removing a portion of cartilage ring as in many standard open tracheotomies, the method of PDT theoretically enters the airway in a tracheal interspace, preventing disruption to the surrounding cartilage. The theory of Hazard et al. [33] may not consider the dynamics of tracheal collapsibility during PDT. The flexibility of the cartilage allows the anterior tracheal surface to be significantly displaced proximally and distally to the intended tracheostoma placement [34]. Injury probably occurs as pressure from the tracheostomy deforms the anterior wall, resulting in macroscopic and microscopic stress fractures of the surrounding cartilage rings.

In specimens and patients with calcification of the trachea, decreased cartilage flexibility is also likely to contribute to comminuted injury. Pressure on a less distensible calcified trachea has a lower fracture threshold. Hotchkiss [1], showed five of six cadaver specimens demonstrated severe cartilage injury beyond the stoma site, including multiple comminutions of two or more rings flanking the stoma. Van Heurn et al. [35] showed that of 12 patients who had undergone autopsy after PDT, 11 had fractures of one or more tracheal rings, 2 of whom had fractured cricoids. These data demonstrate that injury from PDT placement involves more than the area intended for the stoma [1]. In our study, the mucosal injury was found in seven fixed cadavers in PDT arm, while there was no mucosal injury in the MOT arm, which is statistically significant (p 0.008). Also, the cartilage injury was found in eight fixed cadavers in PDT arm, while there was one cartilage injury in the MOT arm, which is statistically significant (p 0.012). While The mucosal injury was found in five fresh cadavers in PDT arm, while there was no mucosal injury in the MOT arm, which is statistically significant (p 0.043). Also, the cartilage injury was found in five fresh cadavers in PDT arm, while there was no cartilage injury in the MOT arm, which is statistically significant (p 0.043).

Our study suggests that a significant degree of damage happens at the time of PDT placement and not after prolonged intubation and ventilatory support. Proponents of PDT placement have stated that the procedure causes minimal disruption of the pretracheal tissue, which then acts as a natural stabilizer for the tracheostomy tube. Relative immobility at the tracheotomy site results in less long-term damage of the cartilage and mucosa [36]. However, our data seems to indicate that greater damage than previously appreciated occurs in the tracheal mucosa and cartilage at the time of PDT placement. A study by Marx et al. [37] suggested that tracheal injury occurring above and below a tracheostomy site is due to prolonged intubation. Although this may be a factor in some cases, our analysis showed a high degree of mucosal damage to the anterior tracheal wall. McFarlane et al. [38] reviewed four cases of stenosis after PDT, all of which had an anterior tracheal wall stenosis. The anterior tracheal location of the stenosis in the study of McFarlane et al. [38] correlates with the area of highest mucosal injury in our study.

A histopathological study by Stoeckli et al. [39] comparing PDT with standard tracheotomy procedures assessed anterior tracheal wall injury in multiple specimens and concluded that PDT creates a higher degree of acute cartilaginous damage than as seen with the standard open tracheotomy procedure.

Percutaneous dilatational tracheostomy under real-time sonographic guidance is a more recent approach that enables precise introducer needle and guidewire insertion, and thus a higher accuracy in placement of a tracheostomy tube is achieved [40, 41].

The decision about which tracheostomy to performs should include an estimate of the potential time for which an individual patient might require a tracheostoma. If the severity of the underlying disease implies a longer period without decannulation, a flap-tracheostomy should be performed. We believe that this would help to minimise the risk of complications, optimise tracheostomy care, and improve the ability of the patient to communicate during rehabilitation. Our personal experience suggests that the creation of a stable and epithelialised tracheostoma may help to avoid inward tearing of the suprastomal anterior tracheal wall, thereby decreasing the risk of developing a tracheal stenosis.

## Conclusions

This study is the first comparison of two commonly established techniques at the anatomical level. Because many patients undergoing PDT have multisystem organ failure and expire before decannulation or weaning from the ventilator, resultant subglottic stenosis either goes unrecognized or does not have time to develop. However, of patients surviving their acute illness after PDT placement, a subset develops subglottic stenosis that may require surgical intervention for successful decannulation. Considering the injury, MOT has better outcome than UPDT.

## Abbreviations

PDT: percutaneous dilation tracheostomy
MOT: mini open techniques
UPDT: ultra percutaneous dilation tracheostomy
CT: computed tomography

## References

[1] Hotchkiss KS, McCaffrey JC (2003). Laryngotracheal injury after percutaneous dilational tracheostomy in cadaver specimens. Laryngoscope.

[2] Jackson C (1909). Tracheostomy. Laryngoscope.

[3] Plummer AL, Gracey DR (1989). Consensus conference on artificial airways in patients receiving mechanical ventilation. Chest.

[4] Walz MK (2002). Tracheostomy. Indications, methods, risks. Anaesthesist.

[5] Sheldon CH, Pudenz RH, Freshwater DB, Crue BL (1955). A new method for tracheostomy. J Neurosurg.

[6] Ciaglia P, Firsching R, Syniec C (1985). Elective percutaneous dilational tracheostomy. A new simple bedside procedure; preliminary report. Chest.

[7] Ciaglia P, Graniero K (1992). Percutaneous dilational tracheostomy: result and long-term follow-up. Chest.

[8] Sue RD, Susanto I (2003). Long-term complications of artificial airways. Clin Chest Med.

[9] Fantoni A, Ripamonti D (1997). A non-derivative, non-surgical tracheostomy: the translaryngeal method. Intensive Care Med.

[10] Griggs WM, Worthley LI, Gilligan JE, Thomas PD, Myburg JA (1990). A simple percutaneous tracheostomy technique. Surg Gynecol Obstet.

[11] Winkler WB, Karnik R, Seelmann O, Havlicek J, Slany J (1994). Bedside percutaneous dilational tracheostomy with endoscopic guidance: experience with 71 ICU patients. Intensive Care Med.

[12] Ciaglia P (1990). Endoscopic guided percutaneous tracheostomy: early results of a consecutive trial. J Trauma.

[13] Norwood S, Vallina VL, Short K, Saigusa M, Fernandez LG, McLarty JW (2000). Incidence of tracheal stenosis and other late complications after percutaneous tracheostomy. Ann Surg.

[14] Massick DD, Yao S, Powell DM, Griesen D, Hobgood T, Allen JN (2001). Bedside tracheostomy in the intensive care unit: a prospective randomized trial comparing open surgical tracheostomy with endoscopically guided percutaneous dilational tracheostomy. Laryngoscope.

[15] Graham JS, Mulloy RH, Sutherland FR, Rose S (1996). Percutaneous versus open tracheostomy: a retrospective cohort outcome study. J Trauma Inj Infect Crit Care.

[16] Benjamin B, Kertesz T (1999). Obstructive suprastomal granulation tissue following percutaneous tracheostomy. Anaesth Intensive Care.

[17] Hill B, Zweng T, Maley R (1996). Percutaneous dilational tracheostomy: report of 356 cases. J Trauma Inj Infect Crit Care.

[18] Levin R, Trivikram L (2001). Cost/benefit analysis of open tracheotomy, in the OR and at the bedside, with percutaneous tracheotomy. Laryngoscope.

[19] Van Heurn LW, Goei R, Ploeg I, Ramsay G, Brink P (1996). Late complications of percutaneous dilational tracheotomy. Chest.

[20] Polderman KH, Spijkstra JJ, De Bree R, Christiaans HM, Gelissen HP, Wester JP (2003). Percutaneous dilational tracheostomy in the ICU: optimal organization, low complication rates, and description of a new complication. Chest.

[21] Bowen CP, Whitney LR, Truwit JD, Durbin CG, Moore MM (2001). Comparison of safety and cost of percutaneous versus surgical tracheostomy. Am Surg.

[22] Kaylie DM, Andersen PE, Wax MK (2003). An analysis of time and staff utilization for open versus percutaneous tracheostomies. Otolaryngol Head Neck Surg.

[23] Krishnan K, Elliot SC, Mallick A (2005). The current practice of tracheostomy in the United Kingdom: a postal survey. Anaesthesia.

[24] Freeman BD, Isabella K, Lin N, Buchman TG (2000). A meta analysis of prospective trials comparing percutaneous and surgical tracheostomy in critically ill patients. Chest.

[25] Cools-Lartigue J, Aboalsaud A, Gill H, Ferri L (2013). Evolution of percutaneous dilatational tracheostomy—a review of current techniques and their pitfalls. World J Surg.

[26] Koitschev A, Simon C, Blumenstock G, Mach H, Graumüller S (2006). Suprastomal tracheal stenosis after dilational and surgical tracheostomy in critically ill patients. Anaesthesia.

[27] Cook PD, Callanan VI (1989). Percutaneous dilational tracheostomy technique and experience. Anaesth Intensive Care.

[28] Hill BB, Zweng TN, Maley RH, Charash WE, Toursarkissian B, Kearney PA (1996). Percutaneous dilational tracheostomy: report of 356 cases. J Trauma.

[29] Friedman Y, Fildes J, Mizock B, Samuel J, Patel S, Appavu S (1996). Comparison of percutaneous and surgical tracheostomies. Chest.

[30] Rosenberg A, Heymann P (1898). Kehlkopf und Luftröhre; Stenosen nach Tracheotomie. Handbuch der Laryngologie und Rhinologie.

[31] Die Thost A, der Stenosen Behandlung, Katz L, Blumenfeld F (1921). Handbuch der Speziellen Chirurgie des Ohresund der Oberen Luftwege.

[32] Dexter JT (1995). A cadaver study appraising accuracy of blind placement of percutaneous tracheostomy. Anaesthesia.

[33] Hazard P, Jones C, Benitone J (1991). Comparative clinical trial of standard operative tracheostomy with percutaneous tracheostomy. Crit Care Med.

[34] Kearney PA, Griffen MM, Ochoa JB, Boulanger BR, Tseui BJ, Mentzer RM (2000). A single-center 8-year experience with percutaneous dilational tracheostomy. Ann Surg.

[35] Van Heurn LW, Theunissen PH, Ramsay G, Brink PR (1996). Pathologic changes of the trachea after percutaneous dilational tracheotomy. Chest.

[36] Schachner A, Ovil J, Sidi J, Avram A, Levy MJ (1990). Rapid percutaneous tracheostomy. Chest.

[37] Marx WH, Ciaglia P, Graniero K (1996). Some important details in the technique of percutaneous dilational tracheostomy via the modified Seldinger technique. Chest.

[38] McFarlane C, Denholm SW, Sudlow CL, Moralee SJ, Grant IS, Lee A (1994). Laryngotracheal stenosis: a serious complication of percutaneous tracheostomy. Anaesthesia.

[39] Stoeckli SJ, Breitbach T, Schmid S (1997). A clinical and histological comparison of percutaneous dilational versus conventional surgical tracheostomy. Laryngoscope.

[40] Chacko J, Gagan B, Kumar U, Mundlapudi B (2015). Real-time ultrasound guided percutaneous dilatational tracheostomy with and without bronchoscopic control: an observational study. Minerva Anestesiol.

[41] Dinh VA, Farshidpanah S, Lu S, Stokes P, Chrissian A, Shah H (2014). Real-time sonographically guided percutaneous dilatational tracheostomy using a long-axis approach compared to the landmark technique. J Ultrasound Med.

